# Congenital mesoblastic nephroma: Clinical analysis of eight cases and a review of the literature

**DOI:** 10.3892/ol.2014.2489

**Published:** 2014-09-01

**Authors:** ZUO-PENG WANG, KAI LI, KUI-RAN DONG, XIAN-MIN XIAO, SHAN ZHENG

**Affiliations:** Department of Pediatric Surgery, Children’s Hospital of Fudan University, Shanghai 201102, P.R. China

**Keywords:** diagnosis, mesoblastic nephroma, treatment

## Abstract

Congenital mesoblastic nephroma (CMN) is a mesenchymal renal tumor. The aim of the present study was to review the clinical characteristics and outcome of CMN in infants. A retrospective file review was conducted of eight cases of CMN treated at the Children’s Hospital of Fudan University between 2004 and 2012. Ultrasound and computerized tomography scans had been performed on all eight patients. Two cases presented with a solid tumor and exhibited pathological features consistent with those of classic CMN, five cases exhibited cystic, hemorrhagic and necrotic characteristics, with calcification and pathology consistent with the cellular variant of CMN and one case presented with a solid tumor, which exhibited pathological features consistent with ceullular CMN. Histology confirmed classic CMN in two patients and cellular CMN in six patients. For surgical intervention, four cases had radical nephrectomy, one case had a half nephrectomy and three cases had tumor enucleation performed. Two cases had received pre-operative chemotherapy, but exhibited no response, and three cases received post-operative chemotherapy. Two patients were lost to follow-up, but the remaining six patients survived to the end of follow-up without further complications. The mean follow-up time was 24.6 months. In conclusion, the differential diagnosis between CMN and Wilms’ tumor is critical. Imaging characteristics are partially correlated with pathological characteristics. Surgery is the main treatment for CMN, but pre-operative chemotherapy is not particularly effective. The efficacy of post-operative chemotherapy requires further investigation, but the prognosis is positive.

## Introduction

Congenital mesoblastic nephroma (CMN) is a mesenchymal renal tumor that was distinguished from Wilms’ tumor (WT) in 1967 (1). CMN is the most frequent type of renal tumor in the neonatal and early infantile period, comprising 3–10% of all childhood renal tumors. CMN has three pathological variants: Classic CMN, the more aggressive cellular CMN and the mixed variant. Classic CMN has a good overall prognosis, but cellular CMN is associated with the potential for malignancy, and is capable of recurrence and metastasis (2). However, surgical resection with nephrectomy is considered an adequate therapy for all subtypes, provided that a complete resection is achieved.

van den Heuvel-Eibrink *et al* (3) reported a five-year event-free survival (EFS) rate of 94% and an overall survival (OS) rate of 96% in infants with CMN. Furtwaengler *et al* (4) reported that patients with the cellular subtype had EFS and OS rates of 85 and 90%, respectively; no recurrences or fatalities were reported among the patients with classic CMN. In the present study, to examine disease features, imaging characteristics, treatment approach and outcome for children with CMN, a retrospective analysis of eight cases of CMN treated between April 2004 and August 2012 at the Children’s Hospital of Fudan University (Shanghai, China) was performed.

## Patients and methods

Eight patients with a diagnosis of CMN attending the Children’s Hospital of Fudan University between 2004 and 2012 were identified, and the clinical charts were reviewed retrospectively to document presentation, treatment and follow-up, subsequent to obtaining approval from the local research ethics committee of the hospital and written informed consent from the parents of each patient.

## Results

### Clinical analysis

The participants included six male and two female patients (Table I). The ages of the patients at diagnosis ranged between five hours and 17 months, with a mean age of 4.4 months. The study included four cases that were diagnosed in the neonatal period. The tumor arose from the left kidney in three patients and from the right kidney in five. Three cases were admitted to the hospital for hematuria, two cases for prenatal diagnosis by ultrasound (US), one case for progressive abdominal distension and two cases for an abdominal mass. The amniotic fluid in all eight cases was normal, but one case exhibited hypertension.

### Imaging analysis

US revealed that three cases were solid tumors, and five had heterogeneity accompanied by cystic areas and either hypoechoic necrosis or hyperechoic calcification. Computerized tomography (CT) revealed similar results, with three cases suggesting the presence of a predominantly solid mass and five patients exhibiting cysts, hemorrhagic, necrosis or calcification components. Of the five cases that had emission CT performed (patients 1,3,5,6 and 8), four exhibited no abnormalities. Case 1 exhibited lesions in the right tibia, which may suggest bone metastases. Combining imaging characteristics and clinical symptoms, seven cases were initially diagnosed as WT and one as neuroblastoma.

### Pathological analysis

All eight patients received surgical treatment: Four cases underwent radical nephrectomy, one case underwent a half nephrectomy and three cases received tumor enucleation. Four of these cases were also treated by lymph node dissection. The pathological findings were as follows: Two cases were diagnosed with classic CMN and six cases with cellular CMN. On gross examination, the CMNs appeared as soft, fleshy tumors, ranging in size between 3×2×2 cm and 20×16×14 cm, with an average volume of 1,310 cm^3^, and the maximum diameter was 20 cm. The cellular CMNs were commonly multicystic with areas of gross hemorrhage, clear fluid accumulations or calcification. Histologically, in classic CMN, uniform fascicles of spindled cells resembling fibroblasts or myofibroblasts are observed, which was also observed in the present study. The mitotic rate is low with relatively abundant collagen. In cellular CMN, fusiform to ovoid high densities of spindle cells impart a sarcomatous appearance. An increased number of mitotic cells are also observed and the cells are more densely arranged (4–6). Seven of the eight cases were also examined using immunohistochemical staining (Table II): Positive vimentin (VIM) and Ki-67 antigen (Ki-67) staining was observed in seven and six cases, respectively; negativity for CD34 antigen (CD34), epithelial membrane antigen (EMA), CKpan (CK), Desmin (DES) and smooth muscle actin (SMA) was observed in six, five, five, four and three cases, respectively.

### Diagnosis

Of the eight cases studied, five were observed to exhibit areas of change involving cysts, necrosis, hemorrhage and calcification in the US and CT examination, and were consistent with cellular CMN. Three cases showed solid or predominantly solid masses, two of which were consistent with classic CMN and one of which was consistent with cellular CMN.

### Treatment and follow-up

Case 1 had received pre-operative chemotherapy with EE-4A (dactinomycin and vincristine). Case 3 relapsed within eight months of a tumor resection and received the DD-4A regimen (dactinomycin, vincristine and doxorubicin) prior to undergoing surgery a second time. However, neither case was responsive to pre-operative chemotherapy, thus the chemotherapeutic regimens were continued subsequent to surgery. Case 8 also received the EE-4A regimen post-surgery. In the three cases with post-operative chemotherapy, one patient was lost to follow-up and the other two cases were resolved without recurrence. In total, with the exception of the two cases lost to follow-up, the other six cases presently exhibit tumor-free survival with a median follow-up time of 24.6 months, the longest follow-up time being 46 months (Table III).

## Discussion

CMN is the most common type of renal tumor in newborns and infants under three months of age, and 90% of cases occur in patients under one year old. The main symptoms at the time of diagnosis are the development of an abdominal mass and hematuria. Increasing numbers of CMN patients are diagnosed in the prenatal period by US (8), including two cases in the present study. The reported male to female ratio is ~1.5:1 and the right kidney to left kidney ratio is ~1:1 (9). In the patient group in the present study, the male to female ratio was 3:1 and the right kidney to left kidney ratio was 5:3. Seven cases were diagnosed in patients under one year old and four were diagnosed in the neonatal period. Despite the small number of cases, the features were consistent with the epidemiological characteristics reported in previous studies. Bayindir *et al* (10) observed that hypertension was present in 70% of CMN patients and the mother is usually reported to have polyhydramnios (11,12). However, only one case in the present study exhibited hypertension and the amniotic fluid levels were normal in all cases. Hypertension and polyhydramnios were uncommon as disease-associated symptoms.

A differential diagnosis between CMN and WT is critical to develop the most effective therapeutic approach. The examination of clinical symptoms and imaging characteristics shows that WT is similar to CMN, particularly the cellular variant, but fewer than 2% patients with WT present at under three months of age. Tumors with congenital syndromes or anomalies, and the presence of bilateral tumors are clearly more suggestive of WT. Electron microscopy provides detailed morphological information that may be applied to achieve the required differential diagnosis. In total, ~24% CMN is reported to be the classic type; cellular variants account for ~66% and mixed variants for ~10% (13). CMN is generally a benign tumor, but occasionally local recurrence occurs and distant metastases have been reported in cellular CMN, with the main site of metastasis the lung. Tumors in the brain, liver, heart, bone and other tissues are extremely rare (14–18).

In the present study, classical CMN accounted for 25% of all cases, cellular CMN accounted for 75% of all cases and no mixed type was observed. Immunohistochemistry aids in performing differential diagnosis; CMN generally exhibits the following results: VIM (+), Ki-67 (+), CD34 (−), EMA (−), CK (−), DES (−) and SMA (−) (4); and this pattern was detected in four out of the seven cases analyzed. Regarding imaging characteristics, Chaudry *et al* (19) reported that the cystic masses, and intratumoral hemorrhagic and necrotic changes commonly appear in cellular CMN. In the present study, if either the US or CT indicated the presence of a solid mass, the tumor was often diagnosed as classic CMN, but if either examination indicated cysts, hemorrhagic necrosis or calcification, the tumor was commonly classified as cellular CMN. In addition, Anderson *et al* (20) analyzed the tumors from 15 CMN cases using reverse transcription polymerase chain reaction and found the existence of the ETV6-NTRK3 fusion gene, which is caused by the (12; 15) (p13; q25) translocation. The three positive cases were all cellular CMN, but the negative cases were of the classic and mixed types, suggesting that ETV6-NTRK3 gene expression levels may be associated with the CMN pathological type. Shared histopathology and translocation gene fusion results support the concept of cellular CMN as the renal form of infantile fibrosarcoma (IFS), while classic CMN is equivalent to infantile fibromatosis (19).

A significant degree of regression in CMN without any treatment, possibly correlated with t (12; 15) (p13; q25) gene translocations, was observed by Whittle *et al* (22), but additional evidence is required for further support. Complete surgical resection is the primary treatment for CMN. The local recurrence rate of CMN is ~5%, commonly due to an incomplete resection (12). England *et al* (9) observed a group of 47 patients with CMN who had been treated by whole nephrectomy and exhibited no recurrence. In the group in the present study, four cases had a whole renal resection, one case received a half nephrectomy and three cases underwent tumor enucleation, with four cases receiving additional lymph node dissection. Case 3 had tumor resection with peritoneal lymph node dissection performed and had a recurrence after eight months, possibly due to the presence of residual tumor tissue. Considering the potential for recurrence, radical nephrectomy is the preferred treatment for CMN.

Case 1, with classical CMN, was initially diagnosed as WT with the possibility of metastases and received an EE-4A treatment regimen. Case 3, with cellular CMN and tumor recurrence after eight months, received a treatment regimen of DD-4A prior to the second surgical treatment. These two cases exhibited no marked response to the pre-operative chemotherapy; the patients received post-operative chemotherapy with the same respective regimens. Case 8, with cellular CMN, received an EE-4A treatment regimen following radical nephrectomy. The decision to administer adjuvant chemotherapy remains controversial and the general preferred treatment for stage I renal tumors is immediate surgery. The likelihood of a renal tumor being non-malignant is markedly reduced with presentation beyond the age of three months (9). Therefore, although surgical risk factors may be perceived, as determined by the condition or tumor characteristics, pre-operative chemotherapy, possibly preceded by biopsy to confirm pathological diagnosis, requires serious consideration.

Pre-operative chemotherapy, with an appropriate dose reduction, has been well-tolerated by young infants (22). If the WT chemotherapeutic regimens are unsuccessful, considering the histological and genetic similarities between CMN and IFS, a sarcoma chemotherapy regimen may be attempted (18). In the patients in the present study, a nine-month old with cellular CMN recurrence and a 17-month old with classic CMN received a WT pre-operative chemotherapy regimen; renal tumors from neither patient had significantly shrunken. Previously, other CMN cases with failed chemotherapy regimens have been reported (7,23). As CMN patients are commonly diagnosed in the first three months of life and are not always sensitive to chemotherapy, routine pre-operative chemotherapy is not recommended and radical surgery is the first choice. If the patient cannot receive surgery, either the WT or sarcoma chemotherapy regimen is accepted as viable treatment (24,25).

Patients older than three months with stage III cellular CMN have been previously found to suffer from a greater recurrence rate than that of younger patients (7). Those with incomplete resection of the tumor, diagnosed by positive lymph node biopsy, and patients with stage III cellular variants, require adjuvant treatment. In the present study, three cases older than three months had stage I–II cellular CMN; two of these patients received post-operative chemotherapy and, upon follow-up, none of the three patients have presented with recurrence. In general, the application of post-operative chemotherapy requires further follow-up observation. Due to complications from chemotherapy, post-operative chemotherapy is not currently recommended for the treatment of the stage I–II cellular variant of CMN; however, a close follow-up of the patients should be conducted. The overall prognosis for CMN is good, but is affected by age and maturity. All six cases in the present study that were not lost to follow-up survived tumor-free.

In conclusion, CMN requires a differential diagnosis from WT and imaging characteristics are partially correlated with pathological characteristics. The US and CT scans revealed cellular CMN as an area with cystic, hemorrhagic and necrotic characteristics and calcification. By contrast, classic CMN was commonly observed as a solid mass. Surgery is the primary treatment, although for patients who cannot receive surgery or patients older than three months with cellular CMN, pre-operative chemotherapy is an option, although the efficacy is uncertain. Patients who have stage III cellular CMN and are aged three months or older at diagnosis may receive post-operative chemotherapy, although the efficacy of this regimen requires further investigation. For patients with stage I–II cellular CMN, chemotherapy is not recommended and the overall prognosis of CMN is fairly good.

## Figures and Tables

**Table I:** Clinical features and pathological findings in eight patients with CMN.

Patient no.	Gender	Age	Location	Reason for admission	Subtype	Initial diagnosis
1	Female	17 m	Left kidney	Abdominal mass	Classic stage IV^a^	WT
2	Male	5 h	Left kidney	Prenatal diagnosis	Classic stage I	WT
3	Male	44 d	Left kidney	Abdominal distension	Cellular stage I	Neuroblastoma
3^b^	Male	9 m	Left kidney	Abdominal mass	Cellular stage II	CMN
4	Male	9 m	Right kidney	Hematuresis	Cellular stage I	WT
5	Male	17 d	Right kidney	Abdominal mass	Cellular stage I	WT
6	Male	13 d	Right kidney	Hematuresis	Cellular stage I	WT
7	Female	11 h	Right kidney	Prenatal diagnosis	Cellular stage I	WT
8	Male	7 m	Right kidney	Hematuresis	Cellular stage I	WT

a Possible subtype;

b Recurrence of case no. 3.

CMN, congenital mesoblastic nephroma; h, hours; d, days; m, months; WT, Wilms’ tumor.

**Table II:** Immunohistochemical staining in CMN patients.

Patient no.	VIM	Ki-67	CD34	EMA	CK	DES	SMA
1	+	−	+	/	+	−	+
2	+	+	−	−	−	/	−
3	/	/	/	/	/	/	/
4	+	+	−	−	−	−	−
5	+	+	−	−	−	−	+
6	+	+	−	−	−	/	/
7	+	+	−	−	−	−	−
8	+	+	−	+	+	/	/
Total	7+	6+, 1−	6−, 1+	5−, 1+	5−, 2+	4−	3−, 2+

CMN, congenital mesoblastic nephroma; VIM, vimentin; Ki-67, Ki-67 antigen; CD34, CD34 antigen; EMA, epithelial membrane antigen; CK, CKpan; DES, Desmin; SMA, smooth muscle actin; +, positive staining; −, negative staining; /, not examined.

**Table III:** Treatment and follow-up of eight CMN cases.

Patient no.	Pre	Post	Surgery	Lymph node dissection	Margin	Follow-up time (months)
1	EE-4A	EE-4A	Nephrectomy	Yes	(−)	Lost
2	/	/	Tumor enucleation	No	(−)	Lost
3	/	/	Tumor enucleation	Yes	(−)	20
3^a^	DD-4A	DD-4A	Tumor resection	Yes	(−)	48
4	/	/	Half nephrectomy	No	(−)	28
5	/	/	Nephrectomy	Yes	(−)	27
6	/	/	Tumor enucleation	No	(−)	22
7	/	/	Nephrectomy	No	(−)	19
8	/	EE-4A	Nephrectomy	Yes	(−)	16

a Recurrence of case no. 3.

CMN, congenital mesoblastic nephroma; pre, preoperative chemotherapy; post, postoperative chemotherapy; EE-4A, dactinomycin and vincristine; DD-4A, dactinomycin, vincristine and doxorubicin; /, not examined; (−), negative.
